# Modern Sociology

**Published:** 1902-10-04

**Authors:** 


### Modern Sociology.

### THE CRÈCHE AND THE SCHOOL.

At what age should a child go to school? The answer to this question may be supposed to be given by that regulation of the Education Department which says that "no attendance is, as a rule, recognised for any scholar under three years of age." Presumably, then, a child's education may begin when he is three years old, and in all conscience that is young enough. Yet the statistics of the Education Department show that in England and Wales there were in 1900-1901 as many as 3,253 children at school who were between the ages of two and three. Between three and four, the numbers rose to 205,744, or 32 per cent. of the total estimated number of children who usually attend elementary schools. Thus it is evident that many parents begin their children's education as soon as they possibly can. No one who knows the class from which the parents of these children belong will think that any intense zeal for education moves them to do so. But the mother is a busy woman. She has a house to attend to, and perhaps one or two children who cannot possibly be sent to school to look after, and it makes her task a little easier if the little ones who are just of an age to be restless and get into mischief are safe within the schoolroom walls. She makes the school a crèche, in short. It might be supposed that teachers would object to this. Of course they cannot protest against the children over three, for these are permitted by the Education Department to attend, and a grant is paid on their attendance. But it might be presumed that no teacher would take in the toddling infants of less than three. But the teachers find that if they do not take in these little ones, there is every chance of an elder sister, who is perhaps making good progress with her school work, being kept at home, just as often as her mother can do so and yet escape prosecution, to look after the younger child. So the teachers submit. It is something to be thankful for that the teaching in the infant rooms in our elementary schools is for the most part kindergarten work. The children are happy at school—happier perhaps than they would be in the vicinity of the overworked and impatient mother aforesaid; but is this herding of so many infants together—a class of a hundred is no exceptional thing—good for their health? And is the kindergarten teaching, however pleasantly it may be given, good for their brains at that stage of their development?

To these questions Dr. Arthur Newsholme, Medical Officer of Health for Brighton, who has been investigating the subject, answers in a decided negative. In an article in *Public Health*, which he calls "A Plea for the Exclusion of Children under Five Years of Age from Public Elementary Schools," he gives strong arguments against it. Although many good teachers avoid the formal teaching of reading, writing, and arithmetic to children under six years of age, this is not universal. Even when the teaching is more strictly "kindergarten" Dr. Newsholme says, "there are strong hygienic objections to putting children of such tender years to word-building, or working patterns with needle and worsted, or, in fact, to any occupation involving trained movements of the muscles of the eye and hand. Excessive strain of vision, resulting in permanent defects of eyesight, often occurs when an attempt is made at these ages to secure accurate manipulations on a small scale involving focussing the eyes on near objects." Moreover, starting so early gives the child no permanent advantage in the educational race. "Children beginning at the later age (*i.e.*, five years) soon overtake the children who begin school attendance at three years of age." This Dr. Newsholme illustrates from the comparative experience of England and Scotland. In Scotland only 2·2 per cent. of the pupils in the elementary schools were under five years of age, while in England the proportion was 10 9. On the other hand, Scotch children remain, as a rule, longer at school than English ones. Only 0 9 of the elementary school children of England and Wales were over 14 years of age, while in Scotland the proportion was 2 5. This means that Scottish parents are willing to forego the advantage of what help their children's earnings can give them until a later period than the law compels, and that Scottish teachers and school authorities think it worth while to continue the education of children for whom they receive no Government grant. And the result, as testified to by a Royal Commission on Elementary Education is that 26 per cent. of the scholars over seven years of age in Scotch schools passed in specific subjects, while this was done by only 2·3 per cent. of the pupils in similar English schools. Perhaps this may help to explain why "the Unspeakable Scot" manages to force his way to the front in England in so many departments of work.

It must be remembered that the education of these children over three years of age and under five, at which age hygienic authorities think it is early enough for a child's education to begin, costs a large amount of public money. Says Dr. Newsholme:—"The average attendance at infant schools being about 72 per cent. of the number on the register, it follows that over £900,000 is spent annually on the attendance at school of children who obtain, according to my contention, no scholastic advantage by such attendance. That money would have served to educate for another year 300,000 boys over 14 years of age, an age when education is most fertile in results, or would have sufficed for the technical education of a smaller but still a large number of scholars, and have enabled those showing the necessary ability to pass on to a university course."

Yet another point Dr. Newsholme brings forward, and one which hygienists throughout the country are bound to take note of. The aggregation of young children in schools gives epidemics of every kind a most favourable chance. That is true of all aggregations of people, but many infectious diseases are most fatal in their result in the earliest years of life. Thus in 1881–1890—the last decade for which complete returns are available—the death-rate for the four most common infectious diseases was, per 100,000 living at all ages:—

| | | | |
|---|---|---|---|
| Mea·les .. .. | under 5, 313; | between 5 & 10, 27; | between 10 & 15, 23 |
| Scarlet Fever .. | " " 167 | " " 76 | " " 15 |
| Diphtheria .. | " " 69 | " " 42 | " " 10 |
| Whooping-cough | " " 119 | " " 13 | " " 0 4 |

From these statistics it is clear that from the point of view of public health the gathering of children together in large schools should be postponed until they have passed the age at which they are most liable to infection, and at which attacks are most likely to end fatally. That this is a real danger may be inferred from the fact that the acceptedly successful way to cut short an epidemic in any school is to close the school. Some of the children may meet and infect each other in their own homes or in the streets, but it will not be such a wholesale infection as arises when a large number of children of the age at which they are most liable to infection are herded together for hours at a time in a schoolroom. Something might perhaps be done to prevent infection by having school doctors, who would visit a school every morning and weed out all suspicious cases. But simpler and cheaper than this is it to forbid the attendance of very young children, especially when it is remembered that their attendance at school, while it causes risks to their physical health, does them no good, but rather harm, in mental development.

